# Traditional Uses, Origins, Chemistry and Pharmacology of *Bombyx batryticatus*: A Review

**DOI:** 10.3390/molecules22101779

**Published:** 2017-10-20

**Authors:** Meibian Hu, Zhijie Yu, Jiaolong Wang, Wenxiang Fan, Yujie Liu, Jianghua Li, He Xiao, Yongchuan Li, Wei Peng, Chunjie Wu

**Affiliations:** 1 College of Pharmacy, Chengdu University of Traditional Chinese Medicine, Chengdu 611137, China; hmbcdtcm@163.com (M.H.); yzj246810@126.com (Z.Y.); helvegps4@126.com (J.W.); fwx13990706098@163.com (W.F.); liu-1567@163.com (Y.L.); lijianghua1413@126.com (J.L.); 2 Chengdu Min-Jiang-Yuan Pharmaceutical Co., Ltd., Chengdu 611000, China; xhe6993@126.com (H.X.); lyc0519@vip.163.com (Y.L.); 3 Key Research Laboratory of Traditional Chinese Medicine Processing Technology, State Administration of Traditional Chinese Medicine of People’s Republic of China, Chengdu 611137, China

**Keywords:** *Bombyx batryticatus*, traditional uses, origin, chemical constituents, pharmacology, toxicity

## Abstract

*Bombyx batryticatus* (*B. batryticatus*), a well-known traditional animal Chinese medicine, has been commonly used in China for thousands of years. The present paper reviewed advances in traditional uses, origin, chemical constituents, pharmacology and toxicity studies of *B. batryticatus*. The aim of the paper is to provide more comprehensive references for modern *B. batryticatus* study and application. In Traditional Chinese Medicine (TCM) culture, drugs containing *B. batryticatus* have been used to treat convulsions, headaches, skin prurigo, scrofula, tonsillitis and fever. Many studies indicate *B. batryticatus* contains various compounds, including protein and peptides, fatty acids, flavonoids, nucleosides, steroids, coumarin, polysaccharide and others. Numerous investigations also have shown that extracts and compounds from *B. batryticatus* exert a wide spectrum of pharmacological effects both in vivo and in vitro, including effects on the nervous system, anticoagulant effects, antitumor effects, antibacterial and antifungal effects, antioxidant effects, hypoglycemic effects, as well as other effects. However, further studies should be undertaken to investigate bioactive compounds (especially proteins and peptides), toxic constituents, using forms and the quality evaluation and control of *B. batryticatus*. Furthermore, it will be interesting to study the mechanism of biological activities and structure-function relationships of bioactive constituents in *B. batryticatus*.

## 1. Introduction

*Bombyx batryticatus* (*B. batryticatus*) is the dried larva of *Bombyx mori* L. (silkworm of 4–5 instars) infected by *Beauveria bassiana* (Bals.) Vuill [1]. It is one of the most popular traditional Chinese medicines, called “Jiangcan” in Chinese vernacular and has been used in China for thousands of years. In addition, it is also widely used in Korea and Japan [2]. *B. batryticatus* is derived from silkworm spontaneously infected by *Beauveria bassiana* originally [3]. Currently, it is mainly produced through artificial breeding techniques by artificial inoculation of *Beauveria bassiana* [4].

*B. batryticatus*, as a common animal medicine in traditional Chinese, Korean, and Japanese medicine systems, has been utilized to treat convulsions, epilepsy, cough, asthma, headaches, skin prurigo, scrofula, tonsillitis, urticarial, parotitis and purpura [2,5,6]. Modern investigations have demonstrated that *B. batryticatus* possesses various pharmacological activities, including effects on nervous system (anticonvulsant effects, antiepileptic effects, and neurotrophic effects), anticoagulant effects, antitumor effects, antibacterial and antifungal effects, antioxidant effects, hypoglycemic effects, as well as other effects [7,8,9]. In addition, it is reported that *B. batryticatus* contains many different constituents including proteins, peptides, fatty acids, flavonoids, nucleosides, steroids, coumarin, polysaccharide and others [7,8,9,10].

In the current review, the advances in traditional uses, origins, chemistry, pharmacology and toxicity of *B. batryticatus* are systematically reviewed. Additionally, the directions and perspectives for future study on *B. batryticatus* are also discussed in the paper.

## 2. Traditional Usages

*B. batryticatus* has been used as a traditional medicine for many centuries in China based on its wide spectrum of biological and pharmacological activities. Traditionally, *B. batryticatus* has commonly been used to treat liver wind with phlegm, convulsion, acute panic of child, tetanus, stroke, fever, headache, sore throat, itchy rubella, as well as mumps [1]. *B. batryticatus* listed firstly in “*Sheng Nong’s herbal classic*”, a famous monograph of Traditional Chinese Medicine (TCM) during the Han Dynasty more than 1000 years ago, and it was described to be useful for the treatment of convulsions of child and skin whitening. Based on “*Ming Yi Bie Lu*” (Liang Dynasty), the main function of *B. batryticatus* was to treat postpartum pain and morbid leucorrhea in women. According to “*Yao Xing Lun*” (Tang Dynasty), *B. batryticatus* was used for the treatment of sweating and uterine bleeding. Subsequently, in “*Xin Xiu Ben Cao*” (Tang Dynasty), another famous TCM monograph, *B. batryticatus* was described as a treatment for furuncle. In addition, according to “*Ben Cao Gang Mu*” (Ming Dynasty), *B. batryticatus* could treat liver wind with phlegm, headache, and furuncle. Later, in “*Yu Qiu Yao Jie*” (Qing Dynasty), *B. batryticatus* was used to treat headache, thoracic obstruction and rubella. In TCM culture, *B. batryticatus* is salty in taste, even in nature and attributive to the liver, lung and stomach meridians [1].

As an animal traditional Chinese medicine, *B. batryticatus* has a little stench smell. In addition, it is reported that *B. batryticatus* has strong side effects on the gastrointestinal tract, and improper use can cause severe allergic reactions [11,12,13]. Therefore, to alleviate its stench smell and alleviate side effects, *B. batryticatus* is commonly processed by stir-frying with bran to a yellowish color [11,12,13]. In addition, the raw *B. batryticatus* and stir-fried *B. batryticatus* are the most common clinically used forms [1]. Although *B. batryticatus* is widely used in TCM, there are limited researches on its side effects and safety evaluations. The Chinese Pharmacopoeia recommends a dose of 5–10 g for *B. batryticatus* [1].

Currently, *B. batryticatus* is a well-known TCM that is used as the main forms of powders, decoctions or infusions for the treatments of convulsion, epilepsy, apoplexy, fever, cough with sputum and other diseases [5,14]. “Chinese Pharmacopoeia”, “Guo Jia Zhong Cheng Yao Biao Zhun”, “Zhong Yao Cheng Fang Zhi Ji”, and “Xin Yao Zhuan Zheng Biao Zhun” revealed 175 prescriptions of Chinese patent drug containing *B. batryticatus*. The present paper summaries prescriptions of Chinese patent drug and decoctions which *B. batryticatus* is the main drug (Table 1).

## 3. Origin

**Figure 1:**
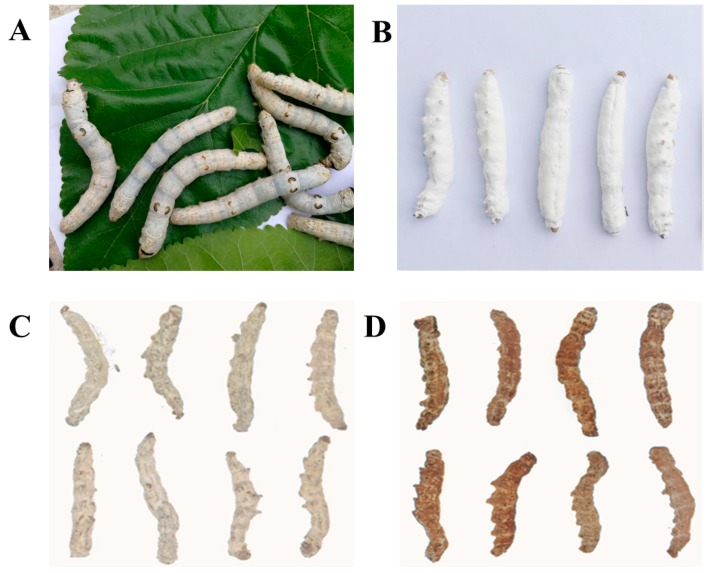
Silkworms (**A**); Silkworms infected by *Beauveria bassiana* (**B**); Bombyx batryticatus (*B. batryticatus*) (**C**); Stir-fried *B. batryticatus* (**D**).

*B. batryticatus* (Figure 1), derived from silkworm spontaneously infected by *Beauveria bassiana*, is the by-product of sericulture, which was described in “*Sheng Nong’s herbal classic*” (Han Dynasty), “*Xin Xiu Ben Cao*” (Tang Dynasty), “*Zheng Lei Ben Cao*” (Song dynasty), “*Tang Ye Ben Cao*” (Yuan dynasty) and “*Ben Cao Pin Hui Jing Yao*” (Ming dynasty). Dictionary of Chinese Pharmacy by Chen (2010) revealed the formation of *B. batryticatus* that before silkworm became moth, it was infected by *Beauveria bassiana* and eventually died [32]. In addition, the lethal mechanism is that when spore of *Beauveria bassiana* infected silkworm, it can secrete chitinase, then dissolve the epidermis and body wall of silkworm and invade into its body, continuously reproduce and eventually cause the death of silkworm. After silkworm is infected by *Beauveria bassiana*, it becomes stiff and its surface covered with white conidias of *Beauveria bassiana* [33].

With development of prevention technology of silkworm diseases, the source of *B. batryticatus* was significantly deficient. Thus, for meeting the market demands, its artificial breeding techniques, namely artificial inoculation of *Beauveria bassiana*, have received more attention and obtained certain development in recent years [4]. The detailed procedure of artificial breeding of *B. batryticatus* is as follows: *Beauveria bassiana* is mixed with warm water and sprayed on silkworms of 4–5 instars; after inoculation for 15–20 min, silkworms are fed with mulberry leaves, and fed every 5.0–6.0 h until they become stiff and white; finally, stiff silkworms are mixed with lime and dried in a ventilated place. The temperature and humidity of the feeding room should be set at 24.0–26.0 °C and 90.0%, respectively [14].

It was recorded that *B. batryticatus* firstly appeared in Yu county, Henan province in Qin and Han Dynasties [3]. During Tang and Song Dynasties, Henan and Shandong were main producing regions of *B. batryticatus* recorded in “*Ben Cao Tu Jing* and *Zheng Lei Ben Cao*”. Later, during Ming and Qing Dynasties, its main regions moved to south area, such as Jiangsu and Zhejiang, which was recorded in “*Ben Cao Chong Yuan*”. Subsequently, Sichuan and Guangdong became the main producing regions of *B. batryticatus*. Currently, the main regions of *B. batryticatus* bred artificially are Sichuan, Jiangsu, Zhejiang, Guangdong, Shandong and Guangxi in China, and the quality of *B. batryticatus* in Sichuan is considered to be the best [34].

## 4. Chemistry

**Figure 2:**
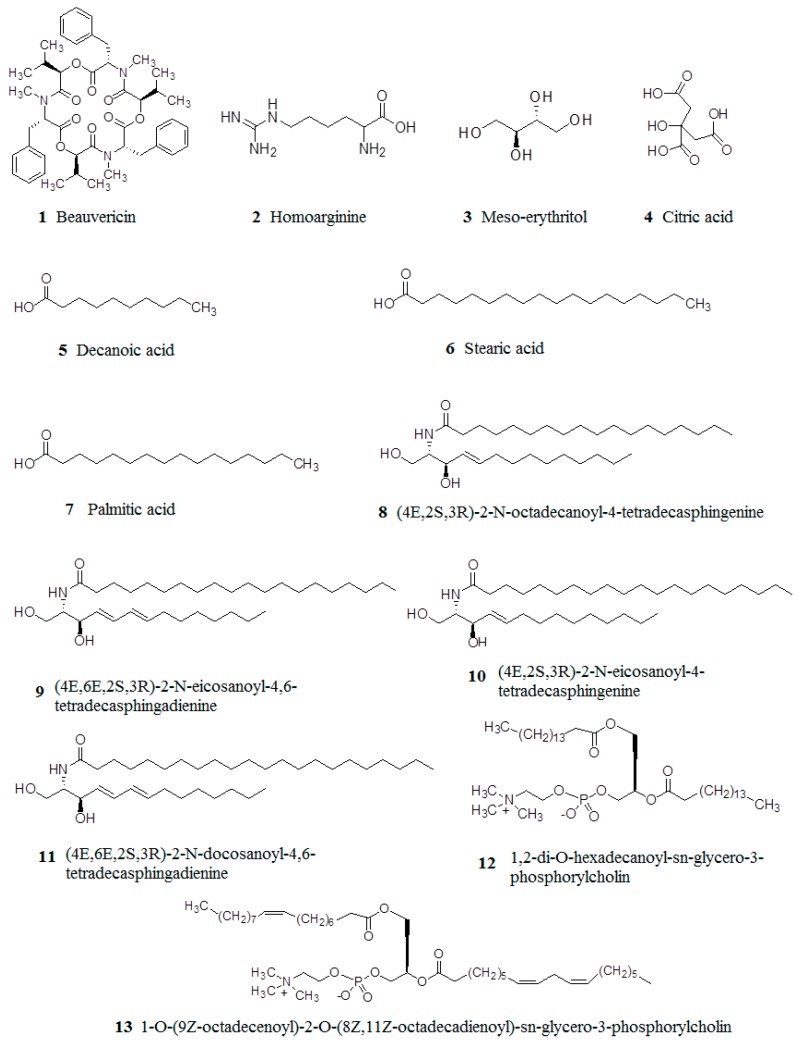

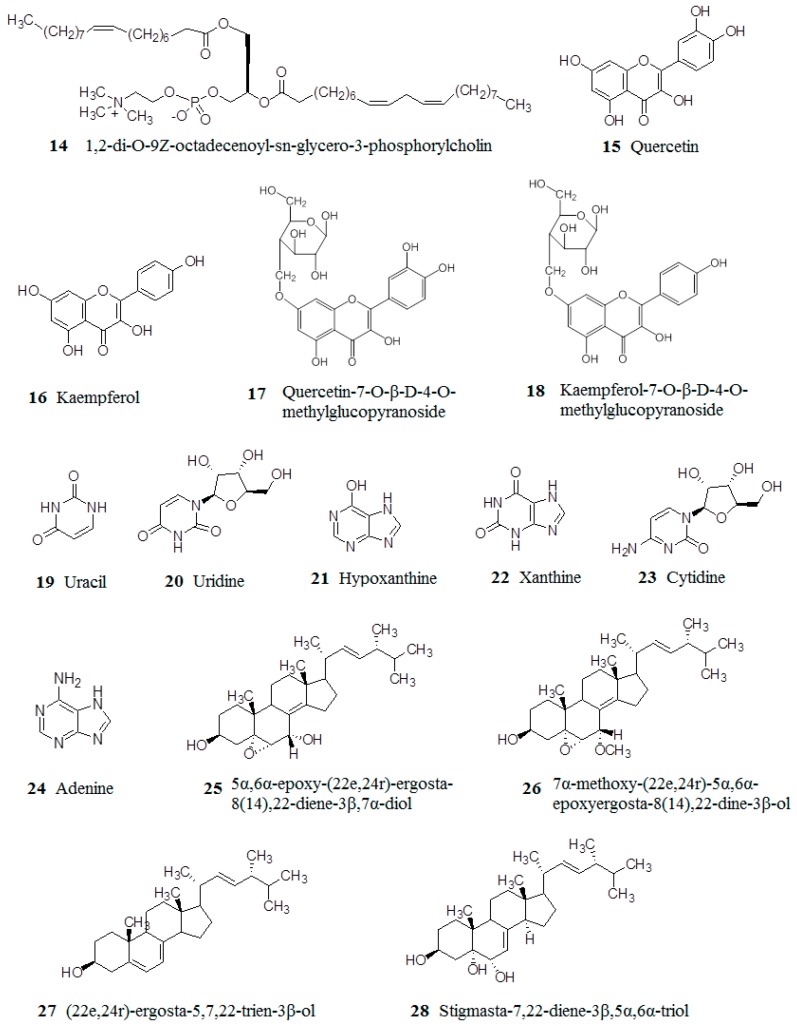

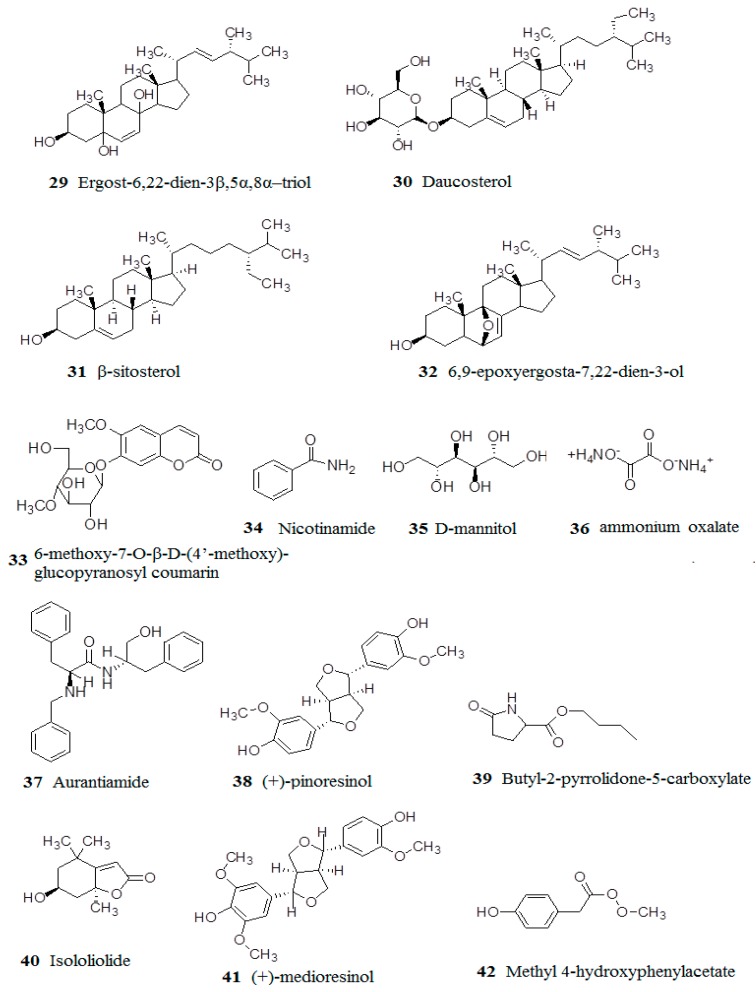
Chemical structures of compounds isolated from *B. batryticatus*.

There are various chemical constituents in *B. batryticatus*, including protein and peptides, fatty acids, flavonoids, nucleosides, steroids, coumarin, polysaccharide and others. In this section, the major chemical constituents and structures of *B. batryticatus* are presented (Table 2 and Figure 2).

### 4.1. Proteins and Peptides

As a traditional animal medicine, the main chemical constituents in *B. batryticatus* are proteins. It is reported that the content of proteins in *B. batryticatus* varies in the range within 43.9–74.3% [45,55]. Currently, some research on peptides in *B. batryticatus* have been reported. BB octapeptide is a novel platelet aggregation inhibitory peptide isolated from *B. batryticatus*, and its molecular mass and the amino acid sequence are 885.0 Da and Asp-Pro-Asp-Ala-Asp-IIe-Leu-Gln, respectively [35]. Beauvericin (**1**), a cyclic three carboxylate peptide, was identified from *B. batryticatus* [36,37,38]. Cyclo(*D*)-Pro-(*D*)-Val, Cyclo(*S*)-Pro-(*R*)-Leu, Cyclo(*D*)-Pro-(*D*)-Ile, Cyclo(*D*)-Pro-(*D*)-Phe and Cyclo-(Ala-Pro), belonging to dipeptide, were also isolated from *B. batryticatus* [36,39]. In 2004, ACIBB were isolated from *B. batryticatus*, whose molecular mass is 1200.0 Da, and it consisted of 7 kinds of amino acids [40]. Later, homoarginine (**2**) was identified from *B. batryticatus* by Cheng et al. (2013a) [41]. Finally, enzymolysis polypeptides by pepsin is studied by Li et al. (2017), and the molecular mass and amino acid number of enzymolysis polypeptide were about 500.0–1000.0 Da and less than 10, respectively [42].

### 4.2. Fatty Acids

Some studies have been carried out to investigate the fatty acids and their derivatives in *B. batryticatus*. Five fatty acids were isolated from *B. batryticatus*: meso-erythritol (**3**), citric acid (**4**), decanoic acid (**5**), stearic acid (**6**) and palmitic acid (**7**) [43,44,45]. Seven derivatives of fatty acids in *B. batryticatus* were identified: (4*E*,2*S*,3*R*)-2-*N*-octadecanoyl-4-tetradecasphingenine (**8**), (4*E*,6*E*,2*S*,3*R*)-2-*N*-eicosanoyl-4,6-tetradecasphingadienine (**9**), (4*E*,2*S*,3*R*)-2-*N*-eicosanoyl-4-tetradecasphingenine (**10**), (4*E*,6*E*,2*S*,3*R*)-2-*N*-docosanoyl-4,6-tetradecasphingadienine (**11**), 1,2-di-*O*-hexadecanoyl-sn-glycero-3-phosphorylcholin (**12**), 1-*O*-(9*Z*-octadecenoyl)-2-*O*-(8*Z*,11*Z*-octadecadienoyl)-sn-glycero-3-phosphorylcholin (**13**) and 1,2-di-*O*-9*Z*-octadecenoyl-sn-glycero-3-phosphorylcholin (**14**) [46,47].

### 4.3. Flavonoids

Flavonoids are common constituents of numerous Chinese medicinal materials. To date, only four flavonoids from *B. batryticatus* have been reported. In 2009, quercetin (**15**) and kaempferol (**16**) were detected in RP-HPLC method and the contents were 0.2 and 0.6 mg/g, respectively [48]. Later, quercetin-7-*O*-β-d-4-*O*-methylglucopyranoside (**17**) and qaempferol-7-*O*-β-d-4-*O*-methylglucopyranoside (**18**) were isolated from *B. batryticatus* [2,36].

### 4.4. Nucleosides

In 1996, four nucleotides were detected in *B. batryticatus* by Li et al. (1996) through HPLC method, including uracil (**19**), uridine (**20**), hypoxanthine (**21**) and xanthine (**22**), and among them, uracil content was highest [47]. Later, in 2003, uracil (**19**), cytidine (**23**) and adenine (**24**) were isolated from *B. batryticatus* by Kwon et al. (2003b) [49]. 

### 4.5. Steroids

Currently, researchers have found and identified many steroids in *B. batryticatus*. Up to now, eight steroids have been identified from *B. batryticatus*: 5α,6α-epoxy-(22*E*,24*R*)-ergosta-8(14),22-diene-3β,7α-diol (**25**), 7α-methoxy-(22*E*,24*R*)-5α,6α-epoxyergosta-8(14),22-dine-3β-ol (**26**), (22*E*,24*R*)-ergosta-5,7,22-trien-3β-ol (**27**), stigmasta-7,22-diene-3β,5α,6α-triol (**28**), ergost-6,22-dien-3β,5α,8α–triol (**29**), daucosterol (**30**), β-sitosterol (**31**) and 6,9-epoxyergosta-7,22-dien-3-ol (**32**) [37,43,50]. 

### 4.6. Coumarin

Limited investigations have been carried out to study the coumarin in *B. batryticatus*. To date, only one coumarin was isolated from *B. batryticatus*: 6-methoxy-7-*O*-β-d-(4′-methoxy)-glucopyranosyl coumarin (**33**) [51].

### 4.7. Polysaccharide

One study of Ying et al. (2015) showed that polysaccharide yield of *B. batryticatus* was about 4.4% and it possessed good antioxidant activity [56]. In addition, BBPW-2 was isolated from *B. batryticatus* and its characteristic was analyzed by Jiang et al. (2014). The results demonstrated that BBPW-2 consisted of β-d-(1 → 2,6)-glucopyranose and β-d-(1 → 2,6)-mannosyl units serving as the backbone, α-d-(1 → 2)-galactopyranose and α-d-(1 → 3)-mannosyl units as branches, and α-d-Manp and β-d-Glcp as terminals [52].

### 4.8. Trace Elements

18 trace elements have been found in *B. batryticatus*, including Al, Fe, Ca, Mg, P, B, Ba, Cu, Cr, La, Mn, Ni, Pb, Sr, Ti, U, Y and Zn. Among them, the contents of Al, Fe, Zn, La and Mn were relatively high [57].

### 4.9. Other Compounds

In addition to the compounds above, some other compounds are also isolated from *B. batryticatus*. In 2003, nicotinamide (**34**) was reported to be isolated from *B. batryticatus* [47]. Then, D-mannitol (**35**) was identified from *B. batryticatus* by Yin et al. (2004a) [43]. Furthermore, it is reported that ammonium oxalate (**36**) was isolated from *B. batryticatus*. [53,54]. Later, in 2015, the following compounds were also found and identified from *B. batryticatus*: aurantiamide (**37**), (+)-pinoresinol (**38**), butyl-2-pyrrolidone-5-carboxylate (**39**), isololiolide (**40**), (+)-medioresinol (**41**) and methyl 4-hydroxyphenylacetate (**42**) were isolated from *B. batryticatus* [39]. 

## 5. Pharmacology

### 5.1. Effects on Nervous System

The characteristic pharmacological activity of *B. batryticatus* is the effects on nervous system, including anticonvulsant and antiepileptic effects, hypnotic effects, neurotrophic effects and others. The beauvericin can significantly prolong latent period of nikethamide-induced and isoniazid-induced convulsion in mice (125.0 and 250.0 mg/kg, s.c.) [58,59]. In addition, β-sitosterol and ergost-6,22-dien-3,5,8-triol were demonstrated to obviously prolong latent period of isoniazid-induced convulsion in mice (125.0 mg/kg, s.c.) [59]. Chloroform fraction of ethanol extract of *B. batryticatus* at dose of 20.0 g/kg showed significant effect on nikethamide-induced convulsion in mice [60]. The results obtained by Yao et al. demonstrated that ethanol extracts of *B. batryticatus* possessed significantly antiepileptic effects on epileptic mice induced by maximal electroshock seizure (MES) and metrazol (MET) in dose-dependent and time-dependent manners [61]. Later, another interesting study reported that ammonium oxalate (30.0 and 60.0 mg/kg) also can inhibit epileptic discharge frequency, amplitude, time and pyramidal cell necrosis in hippocampus region of epileptic rats induced by penicillin [62].

In 2003, it was reported that ethanol extracts of *B. batryticatus* had a significant hypnotic effect on mice (25.0 g/kg, p.o. or 12.5 g/kg, s.c.) and rabbits [63]. The extracts (extracted by water and precipitated by ethanol) of *B. batryticatus* (20.0 g/kg, p.o.) were found to exhibit sedation effect on mice through inhibiting its spontaneous activity [64]. 

In vitro, some compounds (10.0 μM) isolated from *B. batryticatus* were found to exert notable neurotrophic effect by stimulation of NGF (nerve growth factor) synthesis in astrocytes, including (4*E*,2*S*,3*R*)-2-*N*-octadecanoyl-4-tetradecasphingenine, (4*E*,6*E*,2*S*,3*R*)-2-*N*-eicosanoyl-4,6-tetradecasphingadienine, (4*E*,2*S*,3*R*)-2-*N*-eicosanoyl-4-tetradecasphingenine, (4*E*,6*E*,2*S*,3*R*)-2-*N*-docosanoyl-4,6-tetradecasphingadienine, 1-*O*-(9*Z*-octadecenoyl)-2-*O*-(8*Z*,11*Z*-octadecadienoyl)-sn-glycero-3-phosphorylcholine , 1,2-Di-*O*-hexadecanoyl-sn-glycero-3-phosphorylcholine and 1,2-Di-*O*-9*Z*-octadecenoyl-sn-glycero-3-phosphorylcholin [46,47]. Moreover, Bombycis corpus extract (BCE) had a powerful ameliorating effect on neurotoxicity induced by Amyloid-β (Aβ)25–35 in human neuronal cells dose-dependently at the lowest dose of 1.0 μg/mL, and also effectively attenuated the neurotoxic action of NMDA (Nmethyl-d-aspartic acid) [65]. In 2001, another study reported that water extracts of *B. batryticatus* (1.0 × 10^−7^–1.0 × 10^−6^ g/mL) also had significant protective effect against Aβ(25–35) peptide-induced cytotoxicity dose-dependently via inhibiting lipid peroxidation and protecting antioxidative enzymes [66].

### 5.2. Anticoagulant Effect

Anticoagulant effect is another characteristic pharmacological activity of *B. batryticatus*. In 2014, it was reported that BB octapeptide, a novel peptide, can inhibit rabbit platelet aggregation induced by collagen and epinephrine in vitro, with the IC_50_ values of 91.1 and 104.5 μM, respectively [35]. In addition, BB octapeptide also significantly prevented paralysis and death in pulmonary thromboembolism model at doses of 10.0, 30.0 and 50.0 mg/kg, and significantly reduced ferric chloride-induced thrombus formation in rats (5.0, 10.0 and 20.0 mg/kg) [35]. One investigation by Wang et al. (1989) revealed that water extracts of *B. batryticatus* (20.0 mg/mL) could inhibit blood coagulation [67]. 

Zhao et al. (2005) demonstrated that increasing total concentration of ammonium oxalate in water extracts of *B. batryticatus* (33.7–42.3 mg/mL) can prolong TT (thrombase time) [68]. ACIBB (9.0, 18.0 and 36.0 mg/kg, i.v.), belonging to peptide, can significantly inhibit venous thrombosis in rats dose-dependently, by decreasing the contents of Fbg (fibrinogen) and PLg (plasminogen), increasing the activities of tPA (tissue plasminogen activator) and AT-III (antithrombin-III), as well as prolonging APTT (activated partial thromboplastin time), PT (prothrombin time) and TT [69]. Similarly to ACIBB, water extracts of *B. batryticatus* (350.0 mg/kg, i.v.) also possessed fibrinolytic activity and inhibited venous thrombosis [70]. Injection of *B. batryticatus* (150.0 mg/L) was reported that can also inhibit venous thrombosis through increasing tPA activity and decreasing PAI-1 activity [71]. 

### 5.3. Antitumor Effect

Numerous studies have been conducted on antitumor effects of *B. batryticatus* in recent years. *B. batryticatus* possesses significant anti-proliferative effects on human cancer cell lines, such as cervical cancer, liver cancer and gastric cancer [8]. In 2011, it was reported that ethanol extracts of *B. batryticatus* possessed significant anti-cervical cancer effect against HeLa cells at concentrations of 3.0–11.0 mg/mL, and anticancer mechanisms may be associated with induction of apoptosis by down-regulating the expression of Bcl-2 [72]. Another study reported that flavonoids isolated from *B. batryticatus* (50.0–500.0 µg/mL) also showed strong anti-cervical cancer activities through suppressing proliferation of HeLa cells in a concentration-dependent manner [73]. Later, an oligosaccharide BBPW-2 in *B. batryticatus* was demonstrated to have notable anti-cervical cancer (HeLa), anti-liver cancer (HepG2) and anti-breast cancer (MCF-7) activities above the dose of 1.0 mg/mL, and the action mechanism was that BBPW-2-induced cellcycle disruption in the G0/G1 and G2/M phases of early and late apoptotic as well as necrotic cells [52]. In addition, ethanol extract of *B. batryticatus* also had significant anti-cervical cancer activity against HeLa cells with IC_50_ value of 1.7 mg/mL by inducing apoptosis via the regulation of the Bcl-2 and Bax [74]. Recently, it has been reported that ethanol extract of *B. batryticatus* can induce apoptosis of human gastric cancer cells SGC-7901 through upregulating expressions of Bax and P21 and downregulating Bc1-2 expressions with IC_50_ value of 3.2 mg/mL [75]. Another investigation demonstrated that ergosterol, β-Sitosterol and palmitic acid isolated from *B. batryticatus* exerted significant anti-melanoma activities at the lowest concentrations of 0.1, 0.1 and 0.3 mmol/L, respectively [76]. 

### 5.4. Antibacterial and Antifungal Effects

The study of Xiang et al. (2010) revealed that ethanol extracts of *B. batryticatus* possessed antibacterial effect on *Escherichia coli* with MIC (minimal inhibitory concentration) value of 0.6 mg/mL [77]. Another interesting study reported that ethanol extracts of *B. batryticatus* also showed notable antifungal effects on *Colletotrichum gloeosporioides*, *Valsa mali* and leaf cast of *Pericarpium Zanthoxyli* dose-dependently with EC_50_ values of 4.8 × 10^−^^2^, 9.9 × 10^−^^2^ and 7.8 × 10^−^^2^ g/mL, respectively [78]. 

### 5.5. Effects on Viruses

In 2016, one study demonstrated that the supernatant (after ethanol extraction and water precipitation) of *B. batryticatus* possessed antiviral effects against RSV viruses, and the EC_50_ value was 2.7 × 10^−^^2^ g/mL [79]. Interestingly, the research of Zhang et al. (2014) indicated that ethanol extracts of *B. batryticatus* can significantly increase the virulence of HearNPV via inhibition of the ALP (alkaline phosphatase) activity at concentrations of 40.0–80.0 µg/mL [80].

### 5.6. Antioxidant Effect

Reactive oxygen species (ROS) is one of main causes of various types of diseases. In addition, recently, increasing studies have been performed on the antioxidant effect of *B. batryticatus*. In 2013, the study of Jiang et al. (2013) demonstrated that flavonoids isolated from *B. batryticatus* had strong abilities to scavenge DPPH radicals and hydroxyl radicals at concentrations of 5.0 × 10^−^^3^–0.1 mg/mL and 0.1–0.4 mg/mL, respectively [73]. Another investigation reported that methanol extract of *B. batryticatus* possessed notable DPPH radical scavenging, ferric ion-scavenging and lipoxygenase-scavenging activities at the lowest concentrations of 2.0, 8.0 and 4.0 mg/mL, respectively [81]. Later, polysaccharides isolated from *B. batryticatus* possessed a powerful hydroxyl radical-scavenging effect and reducing power at concentrations of 2.5 × 10^−^^2^–0.3 mg/mL [56]. In addition, water extracts of *B. batryticatus* (1.0 × 10^−7^–1.0 × 10^−6^) were reported to possess notable antioxidant effects through inhibiting lipid peroxidation and enhancing SOD activity [66]. 

### 5.7. Other Pharmacological Effects

Increasing investigations suggest that *B. batryticatus* possesses a wide range of other biological activities, such as hypoglycemic effects, anti-fertility effects, improving immune function effects and others. It was reported that flavonoids isolated from *B. batryticatus* can significantly promote proliferation of HEK293 normal human embryo kidney cell lines at concentrations of 50.0–500.0 µg/mL [73]. The study of Zhao et al. (2014) demonstrated that methanol extracts of *B. batryticatus* can inhibit tyrosinase activity at concentrations of 5.0, 10.0, 20.0, 40.0 and 80.0 mg/mL [81]. Another investigation revealed that powder of *B. batryticatus* presented notable hypoglycemic effects in clinical use at the dose of 15.0 g/day for 2 months (p.o.) [82,83]. Additionally, powder of *B. batryticatus* was also reported to relieve headache caused by disturbing-up of *liver Yang* at a dose of 18.0 g/day for 3 days (p.o.) in clinic [84]. In 2002, one interesting study indicated that water extracts of *B. batryticatus* exerted significant anti-fertility effect on mice, and the results showed that water extracts can significantly reduce the weight of ovary, uterus and pregnancy rate in female mice, and increase the weight of testes and seminal vesicles in male mice [85]. Furthermore, another study reported that polysaccharide isolated from *B. batryticatus* can significantly improve immune function via increasing the immune organ weights, improving phagocyte phagocytosis and lymphocyte transformation rate [86]. 

### 5.8. Summary of Pharmacological Effects

*B. batryticatus* possesses a wide spectrum of pharmacological effects, including effects on the nervous system, anticoagulant effects, antitumor effects, antibacterial and antifungal effects, effects on viruses and antioxidant effects, etc. (Table 3). These pharmacological effects show that the extracts and the compounds from *B. batryticatus* can used to prevent or treat certain diseases, in particular convulsions, epilepsy, thrombus and cancer. However, there is not enough systematic data on chemical compounds of *B. batryticatus* and their pharmacological effects.

## 6. Toxicity

Throughout its long history, *B. batryticatus* has been generally considered to be a safe TCM in China [5,14]. However, recent poisoning accidents of *B. batryticatus* were reported by numerous investigations, which is not consistent with traditional understanding of *B. batryticatus* safety. Cheng (2007), Gao (2011), Li et al. (2011a), Liu et al. (2013) reported 46, 216, 425, 248 clinical cases about poisoning accidents of *B. batryticatus*, respectively [87,88,89]. Based on the literature, it can be found that occurrences of poisoning accidents for *B. batryticatus* mainly result from the following reasons: overdose and misuse of *B. batryticatus*, and quality problems caused by non-standard procedure of production and processing [87,88,89,90,91,92,93,94,95]. Furthermore, as a traditional animal medicine, *B. batryticatus* is easily contaminated by aflatoxin, which is regarded as carcinogenic or a teratogenic toxic substance in the procedure of processing, storage and transportation [96]. Therefore, it is urgent and important to standardize methods of production and processing and select the proper doses according to the using form of *B. batryticatus* to avoid adverse reactions and even poisoning. 

It was reported that metabolism of ammonium oxalate in the body can produce ammonia easily, and high content of ammonium oxalate may cause blood ammonia poisoning [97]. The content of ammonium oxalate in *B. batryticatus* is in the range of 5.0–13.0% [53,54]. Thus, overdosing *B. batryticatus* can possibly cause poisoning. Additionally, toxins secreted by *Beauveria bassiana* when using infected silkworm, such as beauvericin, chitosan, chitinase and cellulase, can induce cell death procedurally [9]. Currently, the recognized cause of adverse reactions of *B. batryticatus* is an allergic reaction. Some allogeneic proteins in *B. batryticatus*, can cause sensitization, immune response and even cause metabolic disorder and dysfunction of central nervous system [97]. One investigation demonstrated that proteins secreted by *Beauveria bassiana* can cause adverse effects on mice [97]. However, to date the specific constituents causing adverse reactions or poisoning have not been clarified in *B. batryticatus*. Thus, further studies should be carried out to confirm which constituents are causing side effects or poisoning in *B. batryticatus* and explore corresponding content ranges. 

## 7. Future Perspectives and Conclusions

*B. batryticatus* is one of the most important and frequently used traditional animal medicines, which has been used to treat convulsions, cough, asthma, headaches, skin prurigo, scrofula, tonsillitis and other diseases in China. Recently, *B. batryticatus* has received increasing attention. However, certain aspects still need to be further studied and explored.

There is limited research on bioactive compounds and the mechanism of biological activities of *B. batryticatus*. Thus, it is essential to strengthen research on bioactive compounds, action mechanisms of the bioactive compounds and their structure-function relationships in *B. batryticatus*. Current investigations of *B. batryticatus* mainly focus on its small molecule compounds, but rarely investigate its macromolecular compounds. In addition, as an animal Chinese medicine, the main chemical constituents in *B. batryticatus* are proteins. Therefore, future investigations of *B. batryticatus* could be concentrated on its macromolecular compounds, particularly its proteins and peptides. In addition, mechanisms of biological activities of *B. batryticatus* should be further explored with techniques of modern molecular biology and pharmacology.

Many monographs of TCM record that powder of *B. batryticatus* is used directly in a total of 65 prescriptions where *B. batryticatus* is as the main drug [5,14]. However, in the Pharmacopoeia of the People’s Republic of China of all editions except 1963 edition, the only using form of *B. batryticatus* is decoction. Therefore, further studies should be done to explore which using form (decoction or powder) of *B. batryticatus* is more reasonable and scientific. Furthermore, based on scientific using form of *B. batryticatus*, further studies should be done to analyze reasons of adverse reaction or poisoning caused by *B. batryticatus* and then to establish its safety evaluation system.

Lack of standardized methods of production and processing is another issue of *B. batryticatus*. In the process of production, lime is often used to dry silkworm infected by *Beauveria bassiana* to avoid contamination by miscellaneous bacteria, but lime lacks quality standard and contains a high content of heavy metal and other toxic substances, which seriously affects the quality and safety of *B. batryticatus* [4]. When *B. batryticatus* is processed by stir-frying with bran to a yellowish color, processing degree is mainly evaluated by experience of pharmaceutical worker, which lacks quantifiable indices and is not objective. Thus, it is crucial to standardize the procedure of production and processing using modern technologies for ensuring quality of *B. batryticatus*.

Additionally, as an animal medicine containing complicated compounds, quality evaluation and control of *B. batryticatus* remains challenging for modern researchers. Currently, quality criteria of *B. batryticatus* in the Pharmacopoeia of the People’s Republic of China only includes a description, microscopic identification, check (impurity, contents of water, total ash, acid insoluble ash and aflatoxin) and extract [1], which is inadequate to reflect the holistic quality of *B. batryticatus*. Therefore, it is urgent and important to establish suitable quality evaluation and control systems that can reflect the holistic quality of *B. batryticatus*, such as the fingerprint of the protein or peptide.

In conclusion, this paper provides a comprehensive overview on the traditional uses, chemistry, pharmacology and toxicity of *B. batryticatus*. In addition, this review also provides some trends and perspectives for the future development of *B. batryticatus*.

## Figures and Tables

**Table 1:** The traditional and clinical uses of *B. batryticatus* in China.

Preparation Name	Main Compositions	Traditional and Clinical Uses	References
Qi Zhen Pills	Bombyx Batryticatus, Scorpio, Moschus Artifactus, Cinnabaris, Realgar, Arisaema Cum Bile, Bambusae Concretio Silicea, Crotonis Semen Pulveratum	Relieving convulsion, eliminating sputum, promoting digestion and laxativing, curing acute infantile convulsions, irritability and constipation	“Chinese Pharmacopoeia (2015)”, vol. 1 ^a^
Zhong Feng Hui Chun Pills (Tablets)	Bombyx Batryticatus, Angelicae Sinensis Radix, Carthami Flos, Salviae Miltiorrhizae, Lonicerae Japonicae Caulis, Pheretima, Lycopodii Herba, Scolopendra, Scorpio, Bungarus Parvus	Promoting blood circulation and relaxing tendons, treating apoplexy	“Chinese Pharmacopoeia (2015)”, vol. 1 ^a^
Ru Bi San Jie Capsules	Bombyx Batryticatus, Prunellae Spica, Bupleuri Radix, Rosae Rugosae Flos, Angelicae Sinensis Radix, Ostreae Concha	Activating blood circulation and softening hardness, curing cyclomastopathy	“Chinese Pharmacopoeia (2015)”, vol. 1 ^a^
Bao Ying Powder	Bombyx Batryticatus, Arisaema Cum Bile, Uncariae Ramulus Cum Uncis, Bovis Calculus Artifactus, Scorpio, Margaritifera, Moschus, Typhonii Rhizoma, Gastrodiae Rhizoma, Cicadae Periostracum, Succinum, Saposhnikoviae Radix, Concretio Silica Praeparata, Cinnabaris	Eliminating sputum, relieving convulsion and clearing heat, curing infantile convulsions, fever, excessive phlegm and cough	“Zhong Yao Cheng Fang Zhi Ji”, vol. 6 ^c^
Li Yan Ling Pills	Bombyx Batryticatus, Manis Squama, Eupolyphaga Steleophaga, Ostreae Concha, Scrophulariae Radix	Activating blood circulation and relaxing tendons, relieving sore throat and pain.	“Zhong Yao Cheng Fang Zhi Ji”, vol. 8 ^c^
Li Yan Jie Du Granule	Bombyx Batryticatus, Isatidis Radix, Lonicerae Japonicae Flos, Forsythiae Fructus, Menthae Haplocalycis Herba, Arctii Fructus, Crataegi Fructus, Platycodonis Radix, Isatidis Folium, Scrophulariae Radix, Scutellariae Radix, Rehmanniae Radix, Trichosanthis Radix, Rhei Radix et Rhizoma, Fritillariae Thunbergii Bulbus, Ophiopogonis Radix	Relieving sore throat and clearing heat, curing amygdalitis, pharyngitis and mumps	“Chinese Pharmacopoeia (2015)”, vol. 1^a^
Jia Wei She Dan Chen Pi Pills	Bombyx Batryticatus, Fel Serpentis Siccum, Citri Reticulatae Pericarpium, Pheretima, Cinnabaris, Succinum	Dispelling wind, eliminating sputum and relieving convulsion, treating fever, cough and psychosis	“Zhong Yao Cheng Fang Zhi Ji”, vol. 15 ^c^
Yi Xian Pills	Bombyx Batryticatus, Typhonii Rhizoma, Pinelliae Rhizoma, Scolopendra, Alumen, Cinnabaris	Dispelling wind, eliminating sputum and relieving convulsion, curing epilepsy	“Chinese Pharmacopoeia (2015)”, vol. 1 ^a^
Qian Jin Powder	Bombyx Batryticatus, Scorpio, Bovis Calculus Artifactus, Cinnabaris, Borneolum Syntheticum, Coptidis Rhizoma, Arisaema Cum Bile, Gastrodiae Rhizoma, Glycyrrhizae Radix Et Rhizoma	Clearing heat and relieving convulsion, treating fever and twitch of child	“Zhong Yao Cheng Fang Zhi Ji”, vol. 9 ^c^
Fu Fang Qian Zheng Gao	Bombyx Batryticatus, Typhonii Rhizoma, Pheretima, Scorpio, Chuanxiong Rhizoma, Angelicae Dahuricae Radix, Angelicae Sinensis Radix, Paeoniae Radix Rubra, Saposhnikoviae Radix, Zingiberis Rhizoma Recens, Camphor, Borneolum Syntheticum, Menthol, Thymol	Dispelling wind, activating blood, relaxing tendons and curing apoplexy	“Chinese Pharmacopoeia (2015)”, vol. 1 ^a^
Tai Ji Sheng Jiang Pills	Bombyx Batryticatus, Cicadae Periostracum, Curcumae Longae Rhizoma, Rhei Radix et Rhizoma, Borneolum Syntheticum, Arisaema Cum Bile,	Dispelling wind, clearing heat, eliminating sputum and relieving convulsion, curing fever, twitch, heat phlegm and constipation	“Zhong Yao Cheng Fang Zhi Ji”, vol. 2 ^c^
Wa Wa Ning	Bombyx Batryticatus, Atractylodis, Macrocephalae Rhizoma, Bambusae Concretio Silicea, Uncariae Ramulus Cum Uncis, Glycyrrhizae Radix et Rhizoma, Menthae Haplocalycis Herba, Cinnabaris, Codonopsis Radix, Succinum	Treating cold, fever, spasm, vomiting	“Zhong Yao Cheng Fang Zhi Ji”, vol. 2 ^c^
Niu Huang Xiao Er Powder	Bombyx Batryticatus, Uncariae Ramulus Cum Uncis, Gastrodiae Rhizoma, Scorpio, Picrorhizae Rhizoma, Rhei Radix Et Rhizoma, Arisaema Cum Bile, Fritillariae Thunbergii Bulbus, Bambusae Concretio Silicea, Pinelliae Rhizoma, Citri Exocarpium Rubrum, Talcum, Bovis Calculus Artifactus, Cinnabaris, Moschus Artifactus, Borneolum Syntheticum	Clearing heat, relieving convulsion, eliminating sputum, and dispelling wind, curing cough with sputum and abdominal pain of child	“Zhong Yao Cheng Fang Zhi Ji”, vol. 4 ^c^
Xiao Er Jing Feng Powder	Bombyx Batryticatus, Scorpio, Realgar, Cinnabaris, Glycyrrhizae Radix Et Rhizoma	Relieving convulsion, dispelling wind and treating spasm and coma of child	“Chinese Pharmacopoeia (2015)”, vol. 1 ^a^
Xiao Er Liang You Powder	Bombyx Batryticatus, Menthae Haplocalycis Herba, Gastrodiae Rhizoma, Uncariae Ram, Lus Cum Uncis, Scorpio, Cicadae Periostracum, Bambusae Concretio Silicea, Cinnabaris, Bovis Calculus, Realgar, Succinum	Relieving convulsion, dispelling wind and eliminating sputum, curing infantile convulsions and cough with sputum	“Zhong Yao Cheng Fang Zhi Ji”, vol. 2 ^c^
Jing Feng Pills	Bombyx Batryticatus, Gastrodiae Rhizoma, Atractlodis Rhizoma, Rhei Radix Et Rhizoma, Angelicae Dahuricae Radix, Realgar, Arisaema Cum Bile, Bambusae Concretio Silicea, Bufonis Venenum, Asari Radix et Rhizoma, Scorpio, Cinnabaris	Clearing heat, relieving convulsion and eliminating sputum, treating convulsions, spasm and cough with sputum of child	“Zhong Yao Cheng Fang Zhi Ji”, vol. 4 ^c^
Niu Huang Qian Jin Powder	Bombyx Batryticatus, Scorpio, Bovis Calculus, Cinnabaris, Borneolum Syntheticum, Coptidis Rhizoma, Arisaema Cum Bile, Gastrodiae Rhizoma, Glycyrrhizae Radix Et Rhizoma	Clearing heat and relieving convulsion, curing convulsions, high fever, spasm and cough with sputum of child	“Chinese Pharmacopoeia (2015)”, vol. 1 ^a^
Niu Huang Xiao Er Powder	Bombyx Batryticatus, Arisaema Cum Bile, Pheretima, Uncariae Ramulus Cum Uncis, Aquilariae Lignum Resinatum, Houttuyniae Herba, Bovis Calculus, Borneolum Syntheticum, Margaritifera	Clearing heat, relieving convulsion, dispelling wind and eliminating sputum	“Zhong Yao Cheng Fang Zhi Ji”, vol. 4 ^c^
Niu Huang Bao Long Pills	Bombyx Batryticatus, Bovis Calculus, Arisaema Cum Bile, Bambusae Concretio Silicea, Poria, Succinum, Moschus Artifactus, Scorpio, Realgar, Cinnabaris	Clearing heat, relieving convulsion, dispelling wind and eliminating sputum	“Chinese Pharmacopoeia (2015)”, vol. 1 ^a^
Dian Xian Kang Capsuls	Bombyx Batryticatus, Gastrodiae Rhizoma, Acori Tatarinowii Rhizoma, Arisaema Cum Bile, Fritillariae Cirrhosae Bulbus, Salviae Miltiorrhizae, Radix et Rhizoma, Polygalae Radix, Scorpio, Ophiopogonis Radix, Lophatheri Herba, Zingiberis Rhizoma Recens, Succinum, Ginseng Radix et Rhizoma, Borneolum Syntheticum, Bovis Calculus Artifactus	Relieving convulsion, dispelling wind, and dissipating sputum for resuscitation, treating epilepsy, spasm and vomiting	“Chinese Pharmacopoeia (2015)”, vol. 1 ^a^
Shu Mian Tablets (Capsuls)	Bombyx Batryticatus, Ziziphi Spinosae Semen, Albiziae Cortex, Bupleuri Radix, Paeoniae Radix Alba, Cicadae Periostracum, Albiziae Flos, Junci Medulla	Soothing the liver and calming the heart and tranquilizing the mind, curing insomnia and dizziness	“Xin Yao Zhuan Zheng Biao Zhun”, vol. 81 (33) ^d^
Jin Sang Qing Yin Pills	Bombyx Batryticatus, Scrophulariae Radix, Rehmanniae Radix, Ophiopogonis Radix, Scutellariae Radix, Moutan Cortex, Paeoniae Radix Rubra, Fritillariae Cirrhosae Bulbus, Alismatis Rhizoma, Coicis Semen, Dendrobii Caulis, Menthae Haplocalycis Herba, Sterculiae Lychnophorae Semen, Cicadae Periostracum, Oroxyli Semen, Glycyrrhizae Radix et Rhizoma	Soothing the liver and calming the heart and tranquilizing the mind, curing insomnia	“Zhong Yao Cheng Fang Zhi Ji”, vol. 18 ^c^
Jin Su Dan	Bombyx Batryticatus, Arisaema Cum Bile, Scorpio, Aconiti Coreani Radix, Haematitum, Moschus, Gastrodiae Rhizoma, Borneolum Syntheticum, Olibanum	Dispelling wind and dissipating sputum, and relieving convulsion, treating spasm of child	“Zhong Yao Cheng Fang Zhi Ji”, vol. 6 ^c^
She Xiang Kang Shuang Capsuls	Bombyx Batryticatus, Moschus Artifactus, Saigae Tataricae Cornu, Scorpio, Zaocys, Hirudo, Chuanxiong Rhizoma, Gastrodiae Rhizoma, Rhei Radix Et Rhizoma, Carthami Flos, Arisaema Cum Bile, Spatholobi Caulis, Paeoniae Radix Rubra, Puerariae Thomsonii Radix, Rehmanniae Radix, Astmgali Radix, Lonicerae Japonicae Caulis, Angelicae Sinensis Radix, Trachlospermi Caulis Et Folium, Pheretima, Siegesbeckiae Herba	Promoting blood circulation to remove meridian obstruction, treating apoplexy	“Chinese Pharmacopoeia (2015)”, vol. 1 ^a^
Ling Zhu Powder	Bombyx Batryticatus, Saigae Tataricae Cornu, Shuirongxingzhengzhufen, Bovis Calculus, Cinnabaris, Succinum, Arisaema Cum Bile, Borneolum Syntheticum, Acori Tatarinowii Rhizoma Oil	Clearing heat, and relieving convulsion, curing fever, cough with sputum	“Zhong Yao Cheng Fang Zhi Ji”, vol. 5 ^c^
Dian Xian Ping Tablets	Bombyx Batryticatus, Acori Tatarinowii Rhizoma, Scorpio, Scolopendra, Gypsum Fibrosum, Paeoniae Radix Alba, Magnetitum, Ostreae Concha, Gleditsiae Fructus Abnormalis, Bupleuri Radix	Dissipating sputum to induce resuscitation, clearing heat and relieving convulsion, treating epilepsy	“Guo Jia Zhong Cheng Yao Biao Zhun” vol. of Brain Meridian Limb ^b^
Xiao Er Qing Re Zhen Jing Powder	Bombyx Batryticatus, Coptidis Rhizoma, Arisaema Cum Bile, Bambusae Concretio Silicea, Scorpio, Glycyrrhizae Radix et Rhizoma, Bovis Calculus, Cinnabaris, Borneolum Syntheticum	Clearing heat, and relieving convulsion, curing convulsion, spasm and cough with sputum of child	“Zhong Yao Cheng Fang Zhi Ji”, vol. 4 ^c^
Xiao Er Hua Tang Pills	Bombyx Batryticatus, Bambusae Concretio Silicea, Trichosanthis Radix, Fritillariae Cirrhosae Bulbus, Arisaematis Rhizoma Preparatum, Gastrodiae Rhizoma, Menthae Haplocalycis Herba, Platycodonis Radix, Pinelliae Rhizoma, Acori Tatarinowii Rhizoma, Citri Reticulatae Pericarpium, Cinnabaris	Dispelling wind and dissipating sputum, treating cold, cough with sputum and fever of child	“Zhong Yao Cheng Fang Zhi Ji”, vol. 15 ^c^
Ding Chu Hua Feng Pills	Bombyx Batryticatus, Scorpio, Cicadae Periostracum, Saposhnikoviae Radix, Notopterygii Rhizoma Et Radix, Ephedrae Herba, Platycodonis Radix, Pinelliae Rhizoma, Coptidis Rhizoma, Rhei Radix Et Rhizoma, Glycyrrhizae Radix et Rhizoma, Bovis Calculus Artifactus, Cinnabaris, Moschus, Borneolum Syntheticum	Clearing heat, relieving convulsion, dispelling wind and dissipating sputum, curing convulsion, excessive phlegm and spasm of child	“Zhong Yao Cheng Fang Zhi Ji”, vol. 1 ^c^
Wu she chan yi decoction	Bombyx Batryticatus, Zaocys, Cicadae Periostracum, Vespae Nidus, Senecionis Scandentis Hebra, Dictamni Cortex	Treating acute or chronic eczema	[15]
	Bombyx Batryticatus, Ephedrae Herba, Armeniacae Semen Amarum, Zingiberis Rhizoma, Spirodelae Herba, Dictamni Cortex, Moutan Cortex, Citri Reticulatae Pericarpium, Paeoniae Radix Rubra, Glycyrrhizae Radix et Rhizoma	Treating urticaria	[16]
Shu feng huo xue decoction	Bombyx Batryticatus, Rehmanniae Radix, Kochiae Fructus, Dictamni Cortex, Angelicae Sinensis Radix, Cicadae Periostracum, Xanthii Fructus, Polygoni Multiflori Radix, Praeparata, Lonicerae Japonicae Flos	Treating papular pruritus	[17]
Xiao feng powder	Bombyx Batryticatus, Sophorae Flavescentis Radix, Dictamni Cortex, Rehmanniae Radix, Schizonepetae Herba, Saposhnikoviae Radix, Cicadae Periostracum, Atractylodis Rhizoma, Smilacis Glabrae Rhizoma, Clematidis Armandii Caulis, Angelicae Sinensis Radix	Curing urticaria	[18]
Sheng jiang powder	Bombyx Batryticatus, Cicadae Periostracum, Curcumae Longae Rhizoma, Rhei Radix et Rhizoma	Treating urticarial and pruritus	[19]
	Bombyx Batryticatus, Rhei Radix et Rhizoma, Cicadae Periostracum, Lycii Cortex	Treating diabetes and pruritus	[20]
Wu she san chong decoction	Bombyx Batryticatus, Zaocys, Scorpio, Scolopendra, Sparganii Rhizoma, Curcumae Rhizoma, Manis Squama, Coicis Semen, Smilacis Glabrae Rhizoma, Saposhnikoviae Radix, Bupleuri Radix, Tribuli Fructus, Astragali Radix, Rehmanniae Radix, Glycyrrhizae Radix Et Rhizoma	Curing prurigo nodularis	[21]
Jiang can formula	Bombyx Batryticatus, Violae Herba, Taraxaci Herba, Lonicerae Japonicae Flos, Astmgali Radix, Paeoniae Radix Rubra,	Treating pediatric multiple furuncle	[22]
Xiao ban decoction	Bombyx Batryticatus, Angelicae Dahuricae Radix, Angelicae Sinensis Radix, Chuanxiong Rhizoma, Moutan Cortex, Carthami Flos, Bupleuri Radix, Atractylodis Macrocephalae Rhizoma, Poria, Rehmanniae Radix, Rehmanniae Radix Praeparata, Dioscoreae Rhizoma, Epimedii Folium, Crataegi Fructus, Glycyrrhizae Radix Et Rhizoma	Treating pediatric multiple furuncle	[23]
Xiao ban plaster	Bombyx Batryticatus, Angelicae Dahuricae Radix, Atractylodis, Macrocephalae Rhizoma, Kaempferia Galanga, Carthami Flos, Margarita	Treating chloasma	[23]
Decoction 1	Bombyx Batryticatus, Cmnamomi Mmulus, Paeoniae Radix Alba, Cicadae Periostracum, Astmgali Radix, Atractylodis Macrocephalae Rhizoma, Saposhnikoviae Radix, Glycyrrhizae Radix et Rhizoma, Zingiberis Rhizoma Recens, Jujubae Fructus	Treating rhinallergosis	[24]
Decoction 2	Bombyx Batryticatus, Notoginseng Radix et Rhizoma, Arctii Fructus	Treating verruca plana	[25]
Decoction 3	Bombyx Batryticatus, Cicadae Periostracum	Treating intractable albuminuria and nephropathy	[26,27]
Decoction 4	Bombyx Batryticatus, Lonicerae Japonicae Caulis, Bupleuri Radix, Fritillariae Thunbergii Bulbus, Meretricis Concha, Cyclinae Concha, Cicadae Periostracum, Scorpio, Pheretima, Hirudo, Coicis Semen, Bambusae Caulis In Taenias, Sargassum, Scrophulariae Radix, Paeoniae Radix Rubra, Glycyrrhizae Radix et Rhizoma, Cyathulae Radix, Chaenomelis Fructus, Gypsum Fibrosum, Lycii Cortex	Treating leg pain of the old	[28]
Decoction 5	Bombyx Batryticatus, Lonicerae Japonicae Caulis, Paeoniae Radix Alba, Glycyrrhizae Radix et Rhizoma, Scorpio, Pheretima, Scolopendra, Cicadae Periostracum, Chaenomelis Fructus	Curing trigeminal neuralgia	[28]
Decoction 6	Bombyx Batryticatus, Siphonostegiae Herba, Speranskiae Tuberculatae Herba, Glechomae Herba, Artemisiae Argyi Folium, Croci Stigma	Treating localized scleroderma	[28]
Decoction 7	Bombyx Batryticatus, Astmgali Radix, Atractlodis Rhizoma, Atractylodis, Macrocephalae Rhizoma, Poria, Paeoniae Radix Rubra, Spatholob Caulis, Clematidis Radix et Rhizoma, Coicis Semen, Angelicae Sinensis Radix, Arnebiae Radix, Persicae Semen, Carthami Flos, Sinomenii Caulis, Trachlospermi Caulis et Folium, Piperis Kadsurae Caulis, Galli Gigerii Endothelium Corneum, Glycyrrhizae Radix et Rhizoma Praeparata Cum Melle	Treating dermatomyositis	[28]
Decoction 8	Bombyx Batryticatus, Spatholob Caulis, Violae Herba, Rhei Radix et Rhizoma, Angelicae Sinensis Radix, Bupleuri Radix, Paeoniae Radix Alba, Aurantii Fructus Immaturus, Cicadae Periostracum, Scolopendra, Coicis Semen, Coganargiope, Glycyrrhizae Radix et Rhizoma	Curing chronic appendicitis	[28]
Tian jiang ge gou decoction	Bombyx Batryticatus, Gastrodiae Rhizoma, Puerariae Lobamle Radix, Uncariae Ramulus Cum Uncis, Poria, Pinelliae Rhizoma Praeparatum, Glycyrrhizae Radix et Rhizoma	Relieving convulsion, dispelling wind and treating tremor syndrome	[29]
Chu bi decoction	Bombyx Batryticatus, Scorpio, Schizonepetae Herba, Saposhnikoviae Radix, Notopterygii Rhizoma Et Radix, Angelicae Pubescentis Radix, Glycyrrhizae Radix et Rhizoma	Treating arthromyodynia	[29]
Xiao yin decoction	Bombyx Batryticatus, Sargassum, Laminariae Thallus Eckloniae Thallus, Prunellae Spica, Bambusae Caulis In Taenias, Pinelliae Rhizoma, Sepiae Endoconcha, Glycyrrhizae Radix Et Rhizoma	Treating goiter	[29]
Xiao yong decoction	Bombyx Batryticatus, Violae Herba, Taraxaci Herba, Forsythiae Fructus, Chrysanthemi Flos, Mori Folium, Paeoniae Radix Rubra, Scutellariae Radix, Glycyrrhizae Radix et Rhizoma	Curing multiple furuncle	[29]
Cai ge jie ji jia wei decoction	Bombyx Batryticatus, Bupleuri Radix, Puerariae Lobamle Radix, Angelicae Dahuricae Radix, Gypsum Fibrosum, Notopterygii Rhizoma et Radix, Scutellariae Radix, Platycodonis Radix, Cicadae Periostracum, Rhei Radix Et Rhizoma, Isatidis Folium, Magnoliae Officmalis Cortex	Dispelling wind and clearing heat and treating fever and cold	[30]
Wen dan jia wei decoction	Bombyx Batryticatus, Bambusae Caulis In Taenias, Pinelliae Rhizoma Praeparatum, Cum Alumine, Arisaema Cum Bile, Scutellariae Radix, Ginkgo Folium, Prunellae Spica, Sargassum, Chuanxiong Rhizoma, Ostreae Concha, Alismatis Rhizoma, Fritillariae Thunbergii Bulbus, Cyathulae Radix, Bambusae Concretio Silicea	Treating hypertension	[31]

^a^ Cited from “Chinese Pharmacopoeia”; ^b^ Cited from “Guo Jia Zhong Cheng Yao Biao Zhun”; ^c^ Cited from “Zhong Yao Cheng Fang Zhi Ji”; ^d^ Cited from “Xin Yao Zhuan Zheng Biao Zhun”.

**Table 2:** Chemical constituents isolated from *B. batryticatus*.

Classification	Chemical Component	Molecular Formula	Characteristic Signals or IUPAC Name	References
Peptide	BB octapeptide	---	The molecular mass is 885.0 Da, and the amino acid sequence is Asp-Pro-Asp-Ala-Asp-IIe-Leu-Gln	[35]
	Beauvericin (**1**)	C_45_H_57_N_3_O_9_	(3*S*,6*R*,9*S*,12*R*,15*S*,18*R*)-3,9,15-Tribenzyl-6,12,18-triisopropyl-4,10,16-trimethyl-1,7,13-trioxa-4,10,16-triazacyclooctadecane-2,5,8,11,14,17-hexone	[36,37,38]
	Cyclo(*D*)-Pro-(*D*)-Val	C_10_H_16_O_2_N_2_	---	[39]
	Cyclo(S)-Pro-(*R*)-Leu	C_11_H_18_O_2_N_2_	---	[39]
	Cyclo(*D*)-Pro-(*D*)-Ile	C_11_H_18_O_2_N_2_	---	[39]
	Cyclo(*D*)-Pro-(*D*)-Phe	C_14_H_16_O_2_N_2_	---	[39]
	Cyclo-(Ala-Pro)	C_8_H_12_O_2_N_2_	---	[36]
	ACIBB	---	The molecular mass is 1200.0 Da, and consisting of 7 kinds of amino acids	[40]
	Enzymolysis polypeptides by pepsin	---	The molecular mass were 500.0–1000.0 Da, and the number of amino acid was less than 10	[41]
	Homoarginine (**2**)	C_7_H_16_N_4_O_2_	N^6^-Carbamimidoyl-l-lysin	[42]
Fatty acids	Meso-erythritol (**3**)	C_4_H_10_O_4_	1,2,3,4-Butanetetrol	[43]
	Citric acid (**4**)	C_6_H_8_O_7_	Acide citrique	[44]
	Decanoic acid (**5**)	C_10_H_20_O_2_	Decanoic acid	[45]
	Stearic acid (**6**)	C_18_H_36_O_2_	Octadecanoic acid	[45]
	Palmitic acid (**7**)	C_16_H_32_O_2_	Hexadecanoic acid	[43]
	(4*E*,2*S*,3*R*)-2-*N*-octadecanoyl-4-tetradecasphingenine (**8**)	C_32_H_63_NO_3_	*N*-[(2*S*,3*R*,4*E*)-1,3-Dihydroxy-4-tetradecen-2-yl]octadecanamide	[46]
	(4*E*,6*E*,2*S*,3*R*)-2-*N*-eicosanoyl-4,6-tetradecasphingadienine (**9**)	C_34_H_65_NO_3_	*N*-[(2*S*,3*R*,4*E*,6*E*)-1,3-Dihydroxy-4,6-tetradecadien-2-yl]icosanamide	[46]
	(4*E*,2*S*,3*R*)-2-*N*-eicosanoyl-4-tetradecasphingenine (**10**)	C_34_H_67_NO_3_	*N*-[(2*S*,3*R*,4*E*)-1,3-Dihydroxy-4-tetradecen-2-yl]icosanamide	[46]
	(4*E*,6*E*,2*S*,3*R*)-2-*N*-docosanoyl-4,6-tetradecasphingadienine (**11**)	C_36_H_69_NO_3_	*N*-[(2*S*,3*R*,4*E*,6*E*)-1,3-Dihydroxy-4,6-tetradecadien-2-yl]docosanamide	[46]
	1,2-di-*O*-hexadecanoyl-sn-glycero-3-phosphorylcholin (**12**)	C_40_H_80_NO_8_P	1,2-di-*O*-hexadecanoyl-sn-glycero-3-phosphorylcholin	[47]
	1-*O*-(9*Z*-octadecenoyl)-2-*O*-(8*Z*,11*Z*-octadecadienoyl)-sn-glycero-3-phosphorylcholin (**13**)	C_40_H_72_NO_8_P	1-*O*-(9*Z*-octadecenoyl)-2-*O*-(8*Z*,11*Z*-octadecadienoyl)-sn-glycero-3-phosphorylcholin	[47]
	1,2-di-*O*-9*Z*-octadecenoyl-sn-glycero-3-phosphorylcholin (**14**)	C_41_H_76_NO_8_P	1,2-di-*O*-9*Z*-octadecenoyl-sn-glycero-3-phosphorylcholin	[47]
Flavonoids	Quercetin (**15**)	C_15_H_10_O_7_	2-(3,4-Dihydroxyphenyl)-3,5,7-trihydroxy-4*H*-chromen-4-one	[36,48]
	Kaempferol (**16**)	C_15_H_12_O_6_	3,5,7-Trihydroxy-2-(4-hydroxyphenyl)-4*H*-chromen-4-one	[36,48]
	Quercetin-7-*O*-β-d-4-*O*-methylglucopyranoside (**17**)	C_22_H_23_O_13_	---	[2,36]
	Kaempferol-7-*O*-β-d-4-*O*-methylglucopyranoside (**18**)	C_22_H_25_O_12_	---	[2,36]
Nucleosides	Uracil (**19**)	C_4_H_4_N_2_O_2_	2,4(1*H*,3*H*)-Pyrimidinedione	[43,49]
	Uridine (**20**)	C_9_H_12_N_2_O_6_	4-Hydroxy-1-(β-d-ribofuranosyl)-2(1*H*)-pyrimidinone	[49]
	Hypoxanthine (**21**)	C_5_H_4_N_4_O	3,9-Dihydro-6*H*-purin-6-one	[49]
	Xanthine (**22**)	C_5_H_4_N_4_O_2_	Xanthine	[49]
	Cytidine (**23**)	C_9_H_13_N_3_O_5_	4-Imino-1-(β-d-ribofuranosyl)-1,4-dihydro-2-pyrimidinol	[47]
	Adenine (**24**)	C_5_H_5_N_5_	1*H*-Purin-6-amine	[47]
Steroids	5α,6α-Epoxy-(22*E*,24*R*)-ergosta-8(14),22-diene-3β,7α-diol (**25**)	C_28_H_44_O_3_	5α,6α-Epoxy-(22*E*,24*R*)-ergosta-8(14),22-diene-3β,7α-diol	[50]
	7α-Methoxy-(22*E*,24*R*)-5α,6α-epoxyergosta-8(14),22-dine-3β-ol (**26**)	C_29_H_46_O_3_	7α-Methoxy-(22*E*,24*R*)-5α,6α-epoxyergosta-8(14),22-dine-3β-ol	[50]
	(22*E*,24*R*)-Ergosta-5,7,22-Trien-3β-ol (**27**)	C_28_H_44_O	(22*E*,24*R*)-Ergosta-5,7,22-Trien-3β-ol	[50]
	Stigmasta-7,22-diene-3β,5α,6α-triol (**28**)	C_28_H_46_O_3_	Stigmasta-7,22-diene-3β,5α,6α-triol	[50]
	Ergost-6,22-dien-3β,5α,8α–triol (**29**)	C_27_H_43_O_2_	Ergost-6,22-dien-3β,5α,8α–trio	[43]
	Daucosterol (**30**)	C_35_H_60_O_6_	(3β)-Stigmast-5-en-3-yl-β-d-glucopyranoside	[37,43]
	β-Sitosterol (**31**)	C_29_H_50_O	(3β)-Stigmast-5-en-3-ol	[37,43]
	6,9-Epoxyergosta-7,22-dien-3-ol (**32**)	C_28_H_44_O_2_	(22*E*)-6,9-Epoxyergosta-7,22-dien-3-ol	[37]
Coumarin	6-methoxy-7-*O*-β-d-(4′-methoxy)-glucopyranosyl coumarin (**33**)	C_17_H_20_O_9_	---	[51]
Oligosaccharide	BBPW-2	---	Consisting of β-d-(1 → 2,6)-glucopyranose and β-d-(1 → 2,6)-mannosyl units serving as the backbone, α-d-(1 → 2)-galactopyranose and α-d-(1 → 3)-mannosyl units as branches, and α-d-Manp and β-d-Glcp as terminals	[52]
Other compounds	Nicotinamide (**34**)	C_6_H_6_N_2_O	6-Aminonicotinamide	[47]
	d-Mannitol (**35**)	C_6_H_14_O_6_	d-Mannitol	[43]
	Ammonium oxalate (**36**)	C_2_H_8_N_2_O_4_	Diammonium oxalate	[53,54]
	Aurantiamide (**37**)	C_27_H_28_N_2_O_4_	Nα-Benzoyl-*N*-[(2*S*)-1-hydroxy-3-phenyl-2-propanyl]-l-phenylalaninamide	[39]
	(+)-Pinoresinol (**38**)	C_21_H_24_O_6_	4,4′-(1*S*,3a*R*,4*S*,6a*R*)-Tetrahydro-1*H*,3*H*-furo[3,4-c]furan-1,4-diylbis(2-methoxyphenol)	[39]
	Butyl-2-pyrrolidone-5-carboxylate (**39**)	C_9_H_15_NO_3_	butyl 5-oxo-pyrrolidine-2-carboxylate	[39]
	Isololiolide (**40**)	C_11_H_16_O_3_	(6*S*,7a*S*)-6-hydroxy-4,4,7a-trimethyl-6,7-dihydro-5*H*-1-benzofuran-2-one	[39]
	(+)-Medioresinol (**41**)	C_21_H_24_O_7_	4-[(1*S*,3a*R*,4*S*,6a*R*)-4-(4-Hydroxy-3-methoxyphenyl)tetrahydro-1*H*,3*H*-furo[3,4-c]furan-1-yl]-2,6-dimethoxyphenol	[39]
	Methyl 4-hydroxyphenylacetate (**42**)	C_9_H_10_O_3_	Methyl(4-hydroxyphenyl)acetate	[39]

**Table 3:** Pharmacological effects of *B. batryticatus*.

Pharmacological Effects	Detail	Extracts/Compounds	Minimal Active Concentration/Dose	In Vitro/In Vivo	Reference
**Effects on nervous system**	Anticonvulsant effect	Beauvericin	125.0 mg/kg (s.c.)	in vivo	[58,59]
		β-Sitosterol	125.0 mg/kg (s.c.)	in vivo	[59]
		Ergost-6,22-dien-3,5,8-triol	125.0 mg/kg (s.c.)	in vivo	[59]
		Chloroform fraction of alcohol extract	20.0 g/kg (p.o.)	in vivo	[60]
	Antiepileptic effect	Ethanol extracts	ED_50_ = 18.7 g/kg (p.o.)	in vivo	[61]
		Ammonium oxalate	30.0 mg/kg (i.v.)	in vivo	[62]
	Hypnotic effect	Ethanol extracts	25.0 g/kg (p.o.) or 12.5 g/kg (s.c.)	in vivo	[63]
	Inhibiting spontaneous activity	Water extraction and ethanol precipitation extract	2.0 g/kg (p.o.)	in vivo	[64]
	Neurotrophic effect	(4*E*,2*S*,3*R*)-2-*N*-octadecanoyl-4-tetradecasphingenine	10.0 μM	in vitro	[46]
		(4*E*,6*E*,2*S*,3*R*)-2-*N*-eicosanoyl-4,6-tetradecasphingadienine	10.0 μM	in vitro	[46]
		(4*E*,2*S*,3*R*)-2-*N*-eicosanoyl-4-tetradecasphingenine	10.0 μM	in vitro	[46]
		(4*E*,6*E*,2*S*,3*R*)-2-*N*-docosanoyl-4,6-tetradecasphingadienine	10.0 μM	in vitro	[46]
		1-*O*-(9*Z*-octadecenoyl)-2-*O*-(8*Z*,11*Z*-octadecadienoyl)-sn-glycero-3-phosphorylcholine	10.0 μM	in vitro	[47]
		1,2-Di-*O*-hexadecanoyl-sn-glycero-3-phosphorylcholine	10.0 μM	in vitro	[47]
		1,2-Di-*O*-9*Z*-octadecenoyl-sn-glycero-3-phosphorylcholin	10.0 μM	in vitro	[47]
	Preventing Aβ 25-35 induced neurotoxicity	BCE	1.0 μg/mL	in vitro	[65]
		Water extracts	2.0 × 10^−7^ g/mL	in vitro	[66]
**Anticoagulant effect**	Reducing platelet aggregation induced by collagen and epinephrine	BB octapeptide	IC_50_ = 91.1 μM and 104.5, respectively	in vitro	[35]
	Preventing paralysis and death	BB octapeptide	10.0 mg/kg (i.v.)	in vivo	[35]
	Reducing ferric chloride-induced thrombus formation	BB octapeptide	5.0 mg/kg (i.v.)	in vivo	[35]
	Inhibiting blood coagulation fibrinolytic	Water extracts	20.0 mg/mL	in vitro	[67]
	Prolonging TT	Increasing whole the concentration of ammonium oxalate in water extracts	33. 7 mg/mL	in vitro	[68]
	Inhibiting venous thrombosis and prolonging APTT, PT, TT	ACIBB	9.0 mg/kg (i.v.)	in vivo	[69]
		Water extracts	350.0 mg/kg (i.v.)	in vivo	[70]
	Increasing tPA activity and decreasing PAI-1 activity	Injection	150.0 mg/L	in vitro	[71]
**Antitumor effect**	Anti-cervical cancer (HeLa)	Ethanol extracts	3.0 mg/mL	in vitro	[72]
		Flavonoids	50.0 µg/mL	in vitro	[73]
		BBPW-2	1.0 mg/mL	in vitro	[52]
		ethanol extracts	IC_50_ = 1.6 mg/mL	in vitro	[74]
	Anti-liver cancer (HepG2)	BBPW-2	1.0 mg/mL	in vitro	[52]
	Anti-breast cancer (MCF-7)	BBPW-2	1.0 mg/mL	in vitro	[52]
	Anti-gastric cancer (SGC-7901)	ethanol extracts	IC_50_ = 3.2 mg/mL	in vitro	[75]
	Anti-melanoma activity	Ergosterol	0.1 mmol/L	in vitro	[76]
		β-Sitosterol	0.1 mmol/L	in vitro	[76]
		Palmitic acid	0.3 mmol/L	in vitro	[76]
**Antibacterial and antifungal effects**	Anti-Escherichia coli	Ethanol extracts	MIC = 0.6 mg/mL	in vitro	[77]
	Anti-colletotrichum gloeosporioides	Ethanol extracts	EC_50_ = 4.8 × 10^−2^ g/mL	in vitro	[78]
	Anti-valsa mali	Ethanol extracts	EC_50_ = 9.9 × 10^−2^ g/mL	in vitro	[78]
	Anti-leaf cast of Pericarpium Zanthoxyli	Ethanol extracts	EC_50_ = 7.8 × 10^−2^ g/mL	in vitro	[78]
**Effect on viruses**	Antiviral effect against RSV viruses	Ethanol extraction and water precipitation supernatant	EC_50_ = 2.7 × 10^−2^ g/mL	in vitro	[79]
	Increasing the virulence of HearNPV	Ethanol extracts	40.0 µg/mL	in vitro	[80]
**Antioxidant effect**	Scavenging DPPH	Flavonoids	5.0 × 10^−3^ mg/mL	in vitro	[73]
		Methanol extract	2.0 mg/mL		[81]
	Scavenging hydroxyl radicals	Flavonoids	0.1 mg/mL	in vitro	[73]
		Polysaccharide	2.5 × 10^−2^ mg/mL	in vitro	[56]
		Methanol extract	4.0 mg/mL	in vitro	[81]
	Scavenging ferric ion	Polysaccharide	2.5 × 10^−2^ mg/mL	in vitro	[56]
		Methanol extract	8.0 mg/mL	in vitro	[81]
	Scavenging lipoxygenase	Methanol extract	4.0 mg/mL	in vitro	[81]
	inhibiting lipid peroxidation	Water extracts	2.0 × 10^−7^ g/mL	in vitro	[66]
	Enhancing SOD activity	Water extracts	2.0 × 10^−7^ g/mL	in vitro	[66]
**Other pharmacological effects**	Promoting proliferation of HEK293 cell	Flavonoids	50.0 µg/mL	in vitro	[73]
	Inhibiting tyrosinase activity	Methanol extract	5.0 mg/mL	in vitro	[81]
	Hypoglycemic effect	Crude powder	15.0 g/day (p.o., for 2 months)	in vivo	[82,83]
	Relieving headache	Crude powder	18.0 g/day (p.o., for 3 days)	in vivo	[84]
	Anti-fertility effect	Water extracts	5.0 g/kg (p.o.)	in vivo	[85]
	Improving immune function	Polysaccharide	100.0 mg/kg (p.o.)	in vivo	[86]

## References

[1] Chinese Pharmacopoeia Commission (2015). Pharmacopoeia of the People’s Republic of China Part I.

[2] Kikuchi H., Takahashi N., Oshima Y. (2004). Novel aromatics bearing 4-*O*-methylglucose unit isolated from the oriental crude drug *Bombyx batryticatus*. Tetrahedron Lett..

[3] Ma J.X. (1995). Sheng Nong’s Herbal Classic Jizhu.

[4] Hu M.B., Liu Y.J., Xiao H., Wu C.J., Zhang J.X. (2016). Advance and thinking of researches on artificial culture of *Bombyx batryticatus*. J. Chin. Med. Mater..

[5] Nanjing University of Traditional Chinese Medicine (2006). Traditional Chinese Medicine Dictionary.

[6] Pemberton R.W. (1999). Insects and other arthropods used as drugs in Korean traditional medicine. J. Ethnopharmacol..

[7] Wang J.X., Zhu C.L., Dai H. (1999). The pharmacological research and clinical applications of *Jiangcan* and *Jiangyong*. Lishizhen Med. Mater. Med. Res..

[8] Xu C., Shan S.Y., Liu M., Yang M. (2014). Progress of researches on chemical constituents and pharmacological activities of *Bombyx batryticatus*. China Pharm..

[9] Tian M., Chen F., Yu F. (2015). Progress of research on *Bombyx batryticatus*. Guid. J. Tradit. Chin. Med. Pharmacol..

[10] Yang Q., Liao S.T., Xing D.X., Luo G.Q., Wu F.Q. (2009). Advances in chemical composition of *Bombyx batryticatus* and its identification techniques. Sci. Ser..

[11] Zhang C.W., Peng X.W. (2013). Study on processing technology of *Bombyx batryticatus* stir-fried with bran. J. N. Pharm..

[12] Gong Q.F. (2004). Processing Science of Traditional Chinese Medicine.

[13] Hu M.B., Liu Y.J., Xie D.S., Xiao H., Li Y.C., Wu C.J. (2016). The research on the Maillard reaction in the process of *Bombyx batryticatus* stir-fried with bran. J. Chin. Med. Mater..

[14] State Administration of Traditional Chinese Medicine (1998). Chinese Materia Medica.

[15] Zhang X.J. (1986). Eczema Treat. J. Tradit. Chin. Med..

[16] Zhang Z.L. (2001). Clinical Experience of Skin Disease.

[17] Xu G.X. (1994). Treatments of 100 skin disease cases with modified *Shufeng Huoxue* decoction. J. Sichuan Tradit. Chin. Med..

[18] Wang F.Q., Wang L.Y., Wang L.P., Li Y.N. (1996). Observation on the treatment of allergic dermatosis by using modified *Xiaofeng* powder. China’s Naturop..

[19] Lin S.J. (2002). 4 cases of skin diseases. New. J. Tradit. Chin. Med..

[20] Yao L.Q. (2004). Experience in the treatment of diabetic complications using *Bombyx batryticatus*. Clin. J. Tradit. Chin. Med..

[21] Li H.M. (2005). Clinical observation of 38 prurigo nodularis cases treated with comprehensive TCM. Guid. J. Tradit. Chin. Med. Pharmacol..

[22] Liu H.Y. (1995). Treatments of 33 pediatric multiple cases using *Bombyx batryticatus* powder. Tianjin Tradit. Chin. Med..

[23] Xie Y.L. (2009). The treatment of chloasma using *Bombyx batryticatus*. J. Tradit. Chin. Med..

[24] Zheng J.G., Du W. (2009). Exact effect on allergic rhinitis with *Bombyx batryticatus*. J. Tradit. Chin. Med..

[25] Zhang M., Yang Z.J. (1999). Clinical application of *Bombyx batryticatus*. Inf. Tradit. Chin. Med..

[26] Wang Y.Y., Han Y., He X.Z., Zhang Z.L. (2016). Zhang Zong-li experience in treatment of intractable proteinuria by using *Bombyx batryticatus* and *Cicadae Periostracum*. Hunan J. Tradit. Chin. Med..

[27] Ding Z. (2017). Experience of Professor Ba Yuanming in using TCM herb pairs to treating kidney disease. Acta Chin. Med..

[28] Zhang W.Q., Zhang H.L., Liu R.J., Zhang W.B. (2015). Example for experience cases on the clinical application of *Bombyx batryticatus* of Zhang wen tai aged traditional Chinese medicine doctors. Guangming Tradit. Chin. Med..

[29] Wei Y.B., Zhang Q., Cheng W.P., Jing W. (2015). Clinical applications of *Bombyx batryticatus*. Shandong J. Tradit. Chin. Med..

[30] Liu D.M., Xiong M.B. (2009). *Bombyx batryticatus* has good effect on fever. J. Tradit. Chin. Med..

[31] Zhu L.M., Zhou S.J. (2009). Treatment of hypertension using *Bombyx batryticatus*. J. Tradit. Chin. Med..

[32] Chen C.R. (2010). Dictionary of Chinese Pharmacy.

[33] Wang Y.A. (1982). Research reports of *Beauveria bassiana* and related problems on the production of *Bombyx batryticatus*. J. Xinxiang Teach. Coll..

[34] Wen C.W., Shi L., Zhao Y.Q., Ouyang Z., Zhan Z.L. (2017). Herbal textual research on origin and development of *Bombyx batryticatus*. Lishizhen Med. Mater. Med. Res..

[35] Kong Y., Xu C., He Z.L., Zhou Q.M., Wang J.B., Li Z.Y., Ming X. (2014). A novel peptide inhibitor of platelet aggregation from stiff silkworm, *Bombyx batryticatus*. Peptides.

[36] Huang J.M., Su M.S., Zhang Y.M., Shao F., Yang M., Zhang P.Z. (2017). Chemical Constituents from *Bombyx batryticatus*. J. Chin. Med. Mater..

[37] Li X.H. (2006). Study on the Quality Standard of *Bombyx batryticatus*. Master’s Thesis.

[38] Wang J.H. (2003). The pharmacological research and clinical application of batryticated silkworms and muscardine pupae. Lishizhen Med. Mater. Med. Res..

[39] Huang J.M., Deng H.Y., Cai Y., Li Y., Zhang P.Z., Yang M. (2015). Chemical constituents from *Bombyx batryticatus*. Chin. Tradit. Herb. Drug..

[40] Huang H.Y. (2004). The Study on the Isolation and Purifition about the Anticoagulation of *Bombyx batryticatus*. Master’s Thesis.

[41] Cheng S.M., Li G.Y., Wang H.Y., Huang J., Wang J.H. (2013). Study on chemical constituents of *Bombyx batryticatus*. Mod. Chin. Med..

[42] Li C., Hou L., Yu R.X., Zhang Y.J. (2017). Analysis of enzymolysis polypeptide from *Bombyx batryticatus* by LC–MS. Chem. Anal. Meter..

[43] Yin Z.Q., Ye W.C., Zhao S.X. (2004). Studies on the chemical constituents of *Bombyx batryticatus*. China J. Chin. Mater. Med..

[44] Li J.F., Sun J.M., Zhang H. (2015). *Bombyx batryticatus*’s chemical components and pharmacological activities. Jilin J. Tradit. Chin. Med..

[45] Li D.S., Wang J.H., Hu Z., Li Y.Q. (2003). Main chemical constituents of *Bombyx batryticatus* and its volatile oil analysis. Chem. Bioeng..

[46] Kwon H.C., Lee K.C., Cho O.R., Jung I.Y., Cho S.Y., Kim S.Y., Lee K.R. (2003). Sphingolipids from Bombycis Corpus 101A and their neurotrophic effects. J. Nat. Prod..

[47] Kwon H.C., Jung I.Y., Cho S.Y., Cho O.R., Yang M.C., Lee S.O., Hur J.Y., Kim S.Y., Yang J.B., Lee K.R. (2003). Phospholipids from Bombycis corpus and their neurotrophic effects. Arch. Pharm. Res..

[48] Wang J.J., Zhang M., Zhang X.M. (2009). Content analysis of quercetin and kaempferol in *Bombyx batryticatus* by RP-HPLC. Feed Ind..

[49] Li W., Wen H.M., Zhang A.H., Yu L., Guo R. (1996). The content determinations of 4 kinds of nucleosides and bases in HPLC method. Chin. J. Pharm. Anal..

[50] Cheng S.M., Wang H.Y., Li G.Y., Huang J., Wang J.H. (2013). Study on steroids constituents of *Bombyx batryticatus*. Study on steroids constituents of *Bombyx batryticatus*. J. Shihezi Univ..

[51] Yin Z.Q., Ye W.C., Zhao S.X. (2004). A new coumarin glycoside from *Bombyx batryticatus*. Chin. Tradit. Herb. Drug..

[52] Jiang X., Zhang Z., Chen Y., Cui Z., Shi L. (2014). Structural elucidation and in vitro antitumor activity of a novel oligosaccharide from *Bombyx batryticatus*. Carbohydr. Polym..

[53] Peng X.J., Xu G.M., Li M.J., Jiang X.M. (2006). Determination of oxalic acid ammonium in *Bombyx batryticatus* by HPLC. Cent. S. Pharm..

[54] Huang A.X., Song Y.Y., Yao L.H., Zhou Q., Yu J.Y. (2016). Determination of ammonium oxalate in *Bombyx batryticatus* by ultrasonic extraction-high performance liquid chromatography. Chin. J. Anal. Lab..

[55] Peng X.J., Peng Y.G., Zeng X.Q., Zhao J.G., Jiang X.M., Gao J. (2005). Quantitative analysis of protein and ammonium oxalate in the extract liquide from *Bombyx batryticatus*. Chin. J. Inform. TCM.

[56] Xing D.X., Liao S.T., Luo G.Q., Li L., Xiao Y., Li Q.R., Ye M.Q., Yang Q. (2015). Extraction process optimization and antioxidant activity determination of polysaccharide from *Bombyx batryticatus*. Sci. Seric..

[57] Wei G.Q., Ju G.C., Li H.P., Zhao J. (1995). Analysis of chemical constituents of *Bombyx batryticatus*. J. Jilin Agric. Univ..

[58] Guo X.H., Yan Z.Y., Liu T., Song D.M., Li X.H. (2013). Anticonvulsive activity of three compounds isolated from *Beauveria bassiana*. Chin. J. Exp. Tradit. Med. Form..

[59] Guo X.H., Wu Y.Y., Song D.M., Yan Z.Y., Liu T. (2014). Compounds isolated and purified from chloroform active part of *Bombyx batryticatus* and their anticonvulsive activities. Chin. J. Pharm..

[60] Yan Z.Y., Li X.H., Chen X., Peng C., Liu Y.P., Xiang C. (2006). Aprimary study on anticonvulsant parts of BombyxmoriL. Lishizhen Med. Mater. Med. Res..

[61] Yao H.W., He X.G., He Q.Y., Yang L.H., Ma Y.G. (2006). Comparative study on the anticonvulsant effect of alcohol extract of *Bombyx batryticatus* and *Scolopendra*. Chin. Remed. Clin..

[62] Li W., Xu M.T., Xia Z.Y., Cheng F. (2009). Effects of ammonium oxalate on the epilepsy rat model caused by penicillin. Med. J. Wuhan Univ..

[63] Huang H.Y., Peng X.J., Peng Y.G. (2003). Modern research progress of *Bombyx batryticatus*. J. Hunan Coll. Tradit. Chin. Med..

[64] Hu P.F., Wang J.P., Fan R.P., Chen X.J., Xu Y.X., Pang C.Y. (2005). Study on the sedation of *Bombyx batryicatus*. Lishizhen Med. Mater. Med. Res..

[65] Koo B.S., An H.G., Moon S.K., Lee Y.C., Kim H.M., Ko J.H., Kim C.H. (2003). Bombycis corpus extract (BCE) protects hippocampal neurons against excitatory amino acid-induced neurotoxicity. Immunopharm. Immun..

[66] Kim H.J., Lee W.H., Yoon C.H., Jeong J.C., Nam K.S., Kim H.M., Choo Y.K., Lee M.C., Kim C.H. (2001). Bombycis corpus extract prevents amyloid-beta-induced cytotoxicity and protects superoxide dismutase activity in cultured rat astrocytes. Pharmacol. Res..

[67] Wang J.D., Narui T., Kurata H., Takeuchi K., Hashimoto T., Okuyama T. (1989). Hematological studies on naturally occurring substances. II. Effects of animal crude drugs on blood coagulation and fibrinolysis systems. Chem. Pharm. Bull..

[68] Zhao J.G., Peng X.J., Peng Y.G., Zeng X.Q., Jiang X.M., Zhang Y.H., Lu M.F. (2005). Influence of ammonium oxalate on the action of anticoagalation on stiff silkworm. Lishizhen Med. Mater. Med. Res..

[69] Peng Y.G., Lei T.X., Fu C.Y., Zeng X.Q., Li L.D. (2007). Effect of anticoagulant components in *Bombys batryticatus* on thrombosis. Pharmacol. Clin. Chin. Mater. Med..

[70] Peng Y.G., Li L.D., Deng Y.H. (2001). Study on anti-venous thrombosis and its mechanism in *Bombys batryticatus*. Chin. J. Thromb. Hemo.

[71] Hao X.Y., Su Y., Peng Y.G. (2007). Effect of *Bombyx batryticatus* injection on the balance of fibrinolytic system of human umbilical vascular endothelial cell induced by thrombin. Chin. J. Integr. Tradit. West. Med. Intens. Crit. Care.

[72] Cao J. (2011). Studies on Toxicology of Hela Which Treat with Ethanol Extracts of *Bombyx baticatus*. Master’s Thesis.

[73] Jiang X., Chen Y., Shi L.G. (2013). Optimization of flavonoids extraction from *Bombyx batryticatus* using response surface methodology and evaluation of their antioxidant and anticancer activities in vitro. Food Sci. Biotechnol..

[74] Wu W.P., Cao J., Wu J.Y., Chen H., Wang D. (2015). Anticancer activity of *Bombyx batryticatus* ethanol extract against the human tumor cell line HeLa. Genet. Mol. Res..

[75] Wu J.Y., Sheikho A., Ma H., Li T.C., Zhao Y.Q., Zhang Y.L., Wang D. (2017). Molecular mechanisms of *Bombyx batryticatus* ethanol extract inducing gastric cancer SGC-7901 cells apoptosis. Cytotechnology.

[76] Cheng X.G., Jiang X.H., Liu Z.M., Huang S.Q., Zhou J.Y. (2015). Study on anti-tumor activity of seven chemical constituents of *Bombyx batryticatus*. J. Zhongkai Univ. Agric. Eng..

[77] Xiang L.P., Chai W.L., Wang J., Zheng Y.W., Tang G.H., Lv L., Wang D. (2010). Analysis on antibacterial components from *Bombyx batryticatus* and its antibacterial activity against *Escherichia coli*. J. Northwest Agric. For. Univ..

[78] Chai W.L., Xiang L.P., Wang Y., Zheng Y.W., Tang G.H., Lv L., Wang D. (2009). Inhibitory effect of alcohol extract of *Bombyx batryticatus* on pathogenic fungi of forest. For. Pract. Technol..

[79] Wang Q., Niu W.F., Zhang J.Z., Hou B.S., Liu Y.T., Zhou C.Z. (2016). To determine the effective in vitro antiviral part of *Bombyx Batryticatus*. Shandong J. Tradit. Chin. Med..

[80] Zhang R.F., Ma H., Zhao S.N., Niu M.M., Xing Y.Y., Wang D. (2014). Enhancement of HearNPV by ethanol extact of *Bombyx batryticatus*. J. Northwest Agric. For. Univ..

[81] Zhao Q., Huo L.Q., Jia T.Z. (2014). Effects of Different Processing Methods on in vitro Antioxidant Activity and Inhibiting Capacity for Tyrosinase of *Bombyx batryticatus*. Chin. J. Exp. Tradit. Med. Form..

[82] Ma F.Y. (1990). Treatments of 52 diabetes cases using powder of *Bombyx batryticatus*. Hunan J. Tradit. Chin. Med..

[83] Yang X.J. (2016). Treatments of type 2 diabetes using *Bombyx batryticatus*. China’s Naturop..

[84] Hou X.L., Zhang D.F. (2009). Treatments of headache using *Bombyx batryticatus* singly. J. Tradit. Chin. Med..

[85] Mao X.J., Mao X.P., Xiao Q.C., Jiang Q., Yang X.Y. (2002). Pharmacological study of antifertility on *Bombyx batryticatus*. J. Yunnan Coll. Tradit. Chin. Med..

[86] Jiang X. (2013). Researches on Separation, Purification and Pharmacological Effects of Active Components of *Bombyx batryticatus*. Doctor’s Thesis.

[87] Cheng C.Y. (2007). Clinical treatment of 46 acute poisoning cases caused by *Bombyx batryticatus*. Mod. J. Integr. Tradit. Chin. West. Med..

[88] Gao H. (2011). Clinical observation of toxicity of *Bombyx batryticatus*. Chin. Commun. Doc..

[89] Li D.H., Yan Y.H., Qiu S.S., Guo X.F. (2011). Retrospective analysis of 425 poisoning cases caused by *Bombyx batryticatus*. Strait. Pharm. J..

[90] Liu C.S., Xiang S.H. (2004). Clinical analysis of 248 poisoning cases caused by *Bombyx batryticatus*. Clin. Focus.

[91] Li W., Qian J., Tang W.J. (2011). Analysis of one poisoning case taking powder of *Bombyx batryticatus*. Clin. Misdiag. Misther..

[92] Zhu Y.H. (2011). Emergency nursing to one extrapyramidal reaction cases caused by *Bombyx batryticatus*. Chin. Commun. Doc..

[93] Zhang Z.H. (2011). 2 cases of muscular myoclonus caused by *Bombyx batryticatus*. J. Tradit. Chin. Med..

[94] Feng R.X., Zhang Z.W., Zhang Z.K. (2013). Reports of 2 poisoning cases caused by *Bombyx batryticatus* and countermeasures. J. Tradit. Chin. Med..

[95] Kao X.L. (2013). Report of 1 poisoning cases caused by *Bombyx batryticatus*. J. Zhejiang Univ. Tradit. Chin. Med..

[96] Yang M.H. (2008). Research progress in fungi and mycotoxin infection of medicinal plants and their products. Guizhou Agric. Sci..

[97] Chen Y.T., Cheng D.Q., Lin M.A., Ding Z.S., Pan P.L. (2008). Research on the toxic effect of extracellular proteins secreted by Beauveria bassiana on mice. Chin. Arch. Tradit. Chin. Med..

